# Identification of the *CONSTANS-like* family in *Cymbidium sinense*, and their functional characterization

**DOI:** 10.1186/s12864-023-09884-3

**Published:** 2023-12-18

**Authors:** Youfa Lu, Tengji Li, Xiaolan Zhao, Mingjun Wang, Jiexian Huang, Ziqin Huang, Jaime A. Teixeira da Silva, Jun Duan, Can Si, Jianxia Zhang

**Affiliations:** 1 Guangdong Key Laboratory for Innovative Development and Utilization of Forest Plant Germplasm, College of Forestry and Landscape Architecture, South China Agricultural University, Guangzhou, 510642 China; 2 Key laboratory of South China Agricultural Plant Molecular Analysis and Genetic Improvement, Guangdong Provincial Key Laboratory of Applied Botany, South China Botanical Garden, Chinese Academy of Sciences, Guangzhou, 510650 China; 3 Independent researcher, Miki-cho, Kagawa-ken, Japan

**Keywords:** *Cymbidium sinense*, *CONSTANS-like*, Photoperiod, Flowering time, Functional analysis

## Abstract

**Background:**

*Cymbidium sinense* is an orchid that is typically used as a potted plant, given its high-grade ornamental characteristics, and is most frequently distributed in China and SE Asia. The inability to strictly regulate flowering in this economically important potted and cut-flower orchid is a bottleneck that limits its industrial development. Studies on *C. sinense* flowering time genes would help to elucidate the mechanism regulating flowering. There are very few studies on the genetic regulation of flowering pathways in *C. sinense*. Photoperiod significantly affects the flowering of *C. sinense*, but it was unknown how the *CONSTANS* gene family is involved in regulating flowering.

**Results:**

In this study, eight *CONSTANS-like* genes were identified and cloned. They were divided into three groups based on a phylogenetic analysis. Five representative *CsCOL* genes (*CsCOL3/4/6/8/9*) were selected from the three groups to perform expression characterization and functional study. CsCOL3/4/6/8/9 are nucleus-localized proteins, and all five *CsCOL* genes were expressed in all organs, mainly in leaves followed by sepals. The expression levels of *CsCOL3/4* (group I) were higher in all organs than other *CsCOL* genes. Developmental stage specific expression revealed that the expression of *CsCOL3/4/9* peaked at the initial flowering stage. In contrast, the transcript level of *CsCOL6/8* was highest at the pedicel development stage. Photoperiodic experiments demonstrated that the transcripts of the five *CsCOL* genes exhibited distinct diurnal rhythms. Under LD conditions, the overexpression of *CsCOL3/4* promoted early flowering, and *CsCOL6* had little effect on flowering time, whereas *CsCOL8* delayed flowering of *Arabidopsis thaliana*. However, under SD conditions, overexpression of *CsCOL4/6/8* promoted early flowering and the rosette leaves growth, and *CsCOL3* induced flower bud formation in transgenic *Arabidopsis*.

**Conclusion:**

The phylogenetic analysis, temporal and spatial expression patterns, photoperiodic rhythms and functional study indicate that *CsCOL* family members in *C. sinense* were involved in growth, development and flowering regulation through different photoperiodic pathway. The results will be useful for future research on mechanisms pertaining to photoperiod-dependent flowering, and will also facilitate genetic engineering-based research that uses *Cymbidium* flowering time genes.

**Supplementary Information:**

The online version contains supplementary material available at 10.1186/s12864-023-09884-3.

## Background

In angiosperms, flowering is the most important aspect of reproduction, inducing the occurrence of sexual maturity. In order to regulate flowering time, plants respond to endogenous signals and external environmental stimuli, then decide when to switch from a vegetative to a reproductive state [1, 2]. Photoperiod is an important environmental factor that regulates flowering. *CONSTANS* (*CO*), a key gene that is specific – in terms of its regulation and location-to the central part of the photoperiodic pathway, integrates clock and light signals. The rhythm of *CO* expression, which is regulated by the circadian clock, changes in circadian clock-related gene mutants [3]. *CO* does not directly determine flowering, but might regulate it by controlling the expression of downstream genes *FT* and *SOC1* [4]. *FT* activated the expression of *APETALA1* (*AP1*) and *LEAFY* (*LFY*) in a positive feedback mechanism, ultimately forming a flower meristem [5]. The CO-FT module is a core link in the photoperiodic pathway and is highly conserved in plants [6].

CO is a zinc finger transcription factor that belongs to the BBX protein family and acts as a control center of the photoperiod regulatory network. The CO protein usually contains two conserved domains, a B-box domain near the N-terminus, and a CCT (CO, CO-like, TOC1) domain near the C-terminus [7, 8]. B-box zinc finger motifs may regulate protein–protein interactions [9]. The CCT domain contains a region composed of 43 amino acids and plays an important role in transcriptional regulation and nucleoprotein transport [10]. Many *CO/COL* homologs have been experimentally identified from various plant species. They play critical regulatory roles in the photoperiodic pathway. For example, *AtCO* is induced by photoperiod and promotes flowering in long days (LD) in *Arabidopsis thaliana* [6]. *AtCOL1/2* had little effect on flowering time but their regulation of the period of circadian rhythms was rate-dependent [11]. *AtCOL8/9* delayed flowering in *A. thaliana* in LD [12, 13]. *CO/COL* genes are also involved in photocycle regulatory pathways in monocotyledonous plants, such as maize, barley and rice [14–16]. *OsCO3* represses the transcript levels of *Hd3a* and *FTL* and delays rice heading in short days (SD) [17]. In addition, *CO/COL* genes are also involved in plant hormone signaling, and regulate flowering. AFP2 promotes ABI5 degradation during seed germination, negatively regulates photoperiod-dependent flowering time by modulating the CO signal [18]. Flowering regulation by GA signaling in leaves under LD is mediated through the repression of DELLA by CO [19]. *CO/COL* genes also play an important role in morphological development and stress. *AtCOL3* positively regulated photomorphogenesis, promoted lateral root development, and regulated shoot branching in a daylength-sensitive manner [20]. *AtCOL4* modulated plant tolerance to abiotic stress [21]. *AtCOL7* increased lateral branching and promoted hypocotyl elongation [22]. *Ghd7* delayed rice heading and increased the height and yield of rice in LD [23].

*Cymbidium sinense* is an orchid that is typically used as a potted plant, given its high-grade ornamental characteristics, and is most frequently distributed in China and SE Asia. The inability to strictly regulate flowering in this economically important potted and cut-flower orchid is a bottleneck that limits its industrial development. Studies on *C. sinense* flowering time genes would help to elucidate the mechanism regulating flowering. There are very few studies on the genetic regulation of flowering pathways in *C. sinense*. Photoperiod significantly affects the flowering of *C. sinense*, but it was unknown how the *CO* gene family is involved in regulating flowering. In this study, we isolated and identified eight *CsCOL* (*CONSTANS-like*) genes from the full-length *C. sinense* transcriptome database and analyzed their temporal and spatial expression patterns, as well as photoperiodic rhythms. In addition, subcellular localization and ectopic overexpression of five *CsCOL* genes in *A. thaliana* allowed us to characterize their likely function in photoperiodic flowering. The results will be useful for future research on mechanisms pertaining to photoperiod-dependent flowering, and will also facilitate genetic engineering-based research that uses *Cymbidium* flowering time genes.

## Results

### Identification and phylogenetic analysis of ***COL*** genes in ***C. sinense***

Eight *CONSTANS-like* genes were screened in the *C. sinense* transcriptomic database based on functional annotation of isoforms and analysis of sequence similarity. The eight primer pairs (Table S1.1) were designed and used to clone the ORFs of *CONSTANS-like* genes. The sequences of eight *CsCOL* genes are listed in Table S2. They were named as *CsCOL1* and *CsCOL3*-*9*, respectively. Their GenBank accession numbers are GU168786, OR526963, OR526964, OR526965, OR526966, OR526967, OR526968, OR526969, respectively. The physicochemical properties and subcellular localization of all genes were analyzed. The length of coding sequences (CDS) of the eight *CsCOL* genes ranged between 831 and 1380 bp, and the length of the proteins that they encoded ranged between 227 and 460 amino acids. The range of molecular weights (MWs) spanned from 30.86 to 49.94 kDa, and that of isoelectric points (pIs) spanned from 5.03 to 7.53. Prediction of subcellular localization showed that they were all localized in the nucleus (Table S3).

**Fig. 1:**
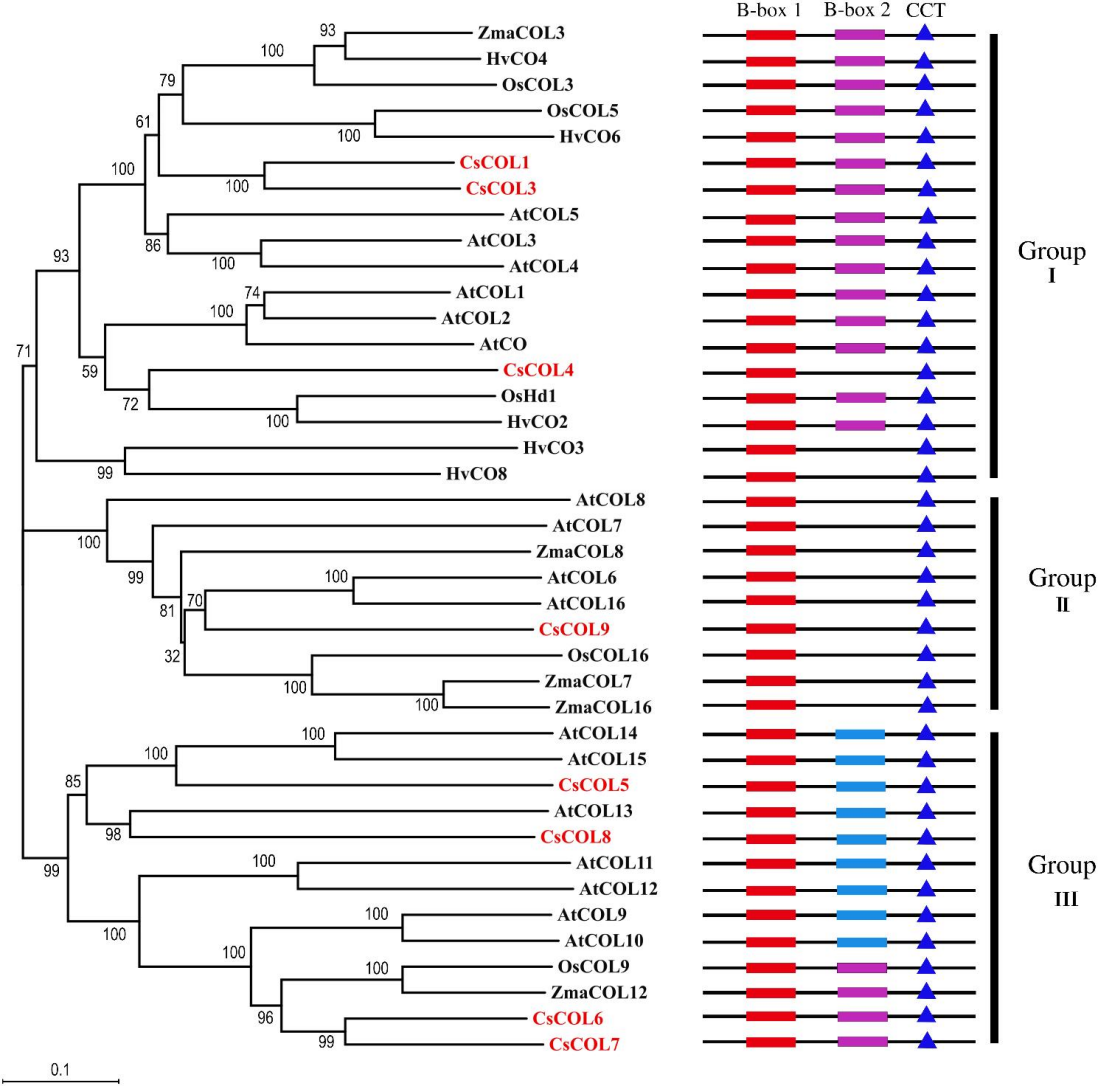
The phylogenetic relationships and conserved domain analysis of CsCOL proteins in *Cymbidium sinense* with COL proteins from *Arabidopsis thaliana* (At), *Oryza sativa* (Os), *Zea mays* (Zm) and *Hordeum vulgare* (Hv). The tree is displayed as a phylogram in which branch lengths are proportional to distance. Bootstrap values for 1000 replicates were used to assess the robustness of the trees. The domain structure of B-box 1 (red rectangles), B-box 2 (purple rectangles), second divergent B-box 2 (blue rectangles) and CTT (dark blue rectangles) of the COL amino acid sequences are shown on the right side. The proteins used for alignment are as follows: *A. thaliana* AtCO (AT5G15840.1), AtCOL1(AT5G15850.1), AtCOL2 (AT3G02380.1), AtCOL3 (AT2G24790.1), AtCOL4 (AT5G24930.1), AtCOL5 (AT5G57660.1), AtCOL6 (AT1G68520.1), AtCOL7 (AT1G73870.1), AtCOL8 (AT1G49130.1), AtCOL9 (AT3G07650.1), AtCOL10 (AT5G48250.1). AtCOL11 (AT4G15250.1), AtCOL12 (AT3G21880.1), AtCOL13 (AT2G47890.1), AtCOL14 (AT2G33500.1), AtCOL15 (AT1G28050.1), AtCOL16 (AT1G25440.1); *O. sativa* OsHd1 (NM_001421538.1), OsCOL3 (NM_015770369.2), OsCOL5 (XM_015785723.2), OsCOL9 (XM_026022873.1), OsCOL16 (XM_015767173.2); *Z. mays* ZmaCOL3 (GRMZM2G012717_P01), ZmaCOL7 (GRMZM2G041991_P01), ZmaCOL8 (GRMZM2G095598_P01), ZmaCOL12 (GRMZM2G042198_P01), ZmaCOL16 (GRMZM2G013398_P04); *H. vulgare* HvCO2 (AAM74064.1), HvCO3 (AAM74068.1), HvCO4 (AAM74069.1), HvCO6 (AAL99268.1), HvCO8 (AAL99270.1)

An amino acid-based phylogenetic tree, which was constructed with MEGA11, was used to assess the evolutionary relationships of the eight *CsCOL* genes against the *COL* genes of other plants, namely *A. thaliana*, *Oryza sativa*, *Zea mays*, and *Hordeum vulgare*. Based on this phylogenetic analysis (Fig. 1), the amino acid sequences of eight CsCOL proteins were classified into three groups according to the number and structure of conserved B-box domains. CsCOL1/3/4 were clustered in Group I. CsCOL1/3 contained two B-boxes and a CCT domain, similar to AtCO and AtCO1-5. CsCOL1/3 formed a sister group and displayed 69% identity with each other. CsCOL3 also shared 36% and 34% identity with AtCO from *A. thaliana* and OsHd1 from *O. sativa*, respectively. CsCOL4, similar to HvCO3/8, only possessed a B-box and a CCT domain, but was classified into Group I. CsCOL4 was only 30% and 24% identical to CsCOL1 and CsCOL3, respectively. However, CsCOL4 shared a high identity with AtCO (33%) and OsHd1 (32%). Among the eight *CsCOL* genes, only CsCOL9 was found in Group II. CsCOL9 contained one B-box domain and a CCT domain, similar to AtCOL6-8 and AtCOL16. CsCOL5-8, which were clustered in Group III, contained a normal B-box domain, a divergent B-box domain and a CCT domain, similar to AtCOL9-15. CsCOL6/7 formed a sister group that was closely related to OsCOL9, and AtCOL9/10. CsCOL6/7 showed 65% identity with each other, also shared a high identity with OsCOL9 (61%) and AtCOL9 (63%). Most *CO/COL* homologs in the same group possessed the same protein domain structure. Apart from CsCOL4 in Group I, this phylogenetic classification of CsCOL6/7, OsCOL9 and ZmaCOL12 in Group III were also different from the classification based on differences in the B-box domain, containing two B-boxes, similar to the protein domain structure in Group I (Fig. 1). This diversity of *CsCOL* genes may indicate the existence of functional genetic divergence in *C. sinense.*

**Fig. 2:**
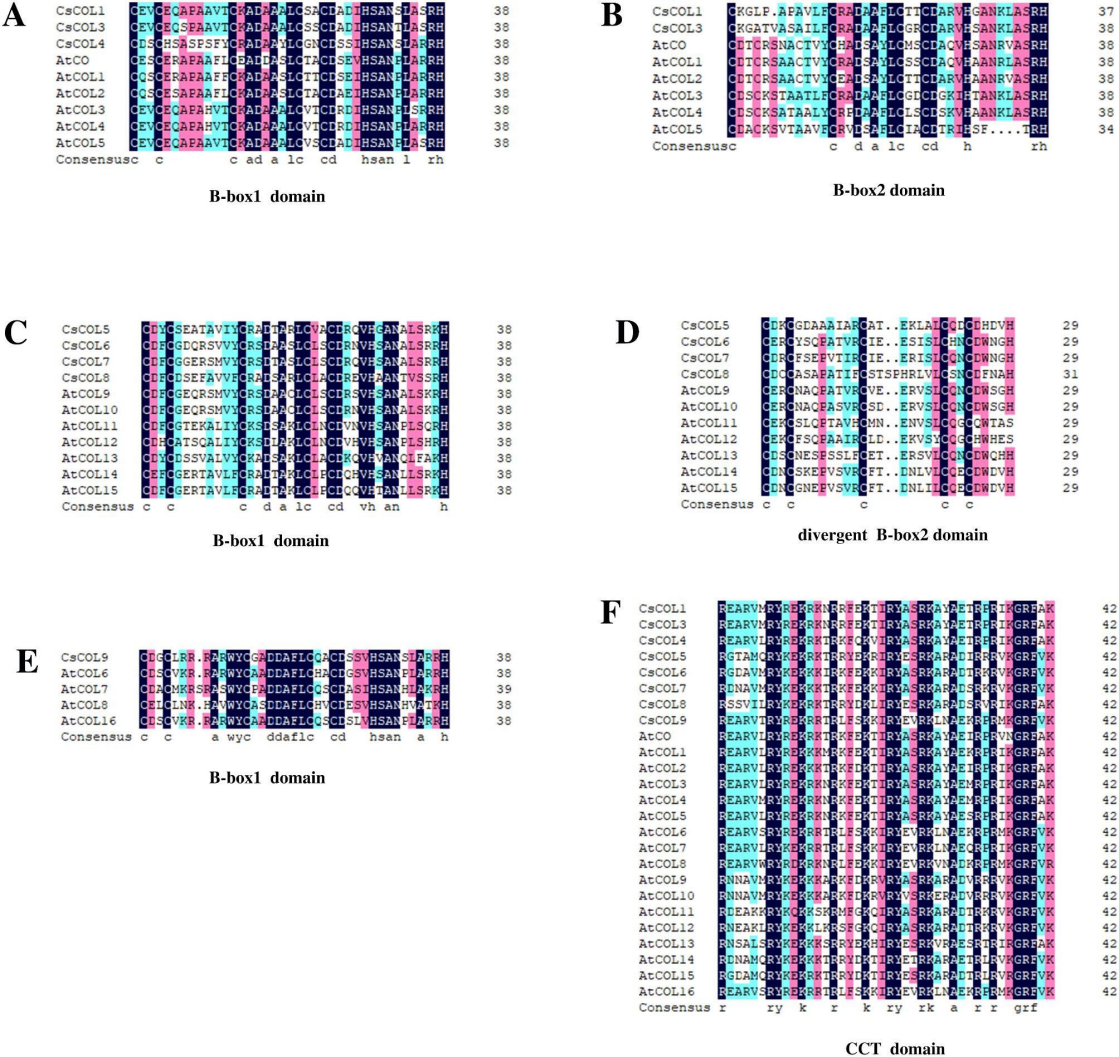
The conserved domain alignment of CsCOL and AtCOL proteins. (**A**) Alignment of the B-box1 domains of CsCOL1/3/4, AtCO and AtCOL1–5 in Group I. (**B**) Alignment of the B-box2 domains of CsCOL1/3/4, AtCO and AtCOL1–5 in Group I. (**C**) Alignment of the B-box1 domains of CsCOL5–8 and AtCOL9–15 in Group III. (**D**) Alignment of the divergent B-box2 domains of CsCOL5–8 and AtCOL9–15 in Group III. (**E**) Alignment of the B-box1 domains of CsCOL9 and AtCOL6/7/8/15 in Group II. (**F**) Alignment of the CCT domains of all the CsCOL and AtCOL proteins

To further analyze the differences of *COL* homologs in the three groups, the amino acid sequences in conserved B-boxes and CCT domains of eight CsCOL and 17 AtCOL proteins were aligned (Fig. 2). The alignment indicated that B-box1 and B-box2 domains displayed 82% and 73% identity between CsCOL1/3 and AtCOL proteins in Group I, respectively (Fig. 2A and B). The consensus sequence of B-box1 was C-X2-C-X8–C-X-A-D-X-A-X-L-C-X2-C-D-X3-H-S-A-N-X-L-X2-R-H, and 17 out of 38 (44.7%) amino acids were fully conserved (Fig. 2A). The consensus sequence of B-box2 was C-X11-C-X2-D-X-A-X-L-C-X2-C-D-X3-H-X7-R-H, and 11 out of 38 (28.9%) amino acids were fully conserved (Fig. 2B). The B-box1 and divergent B-box2 domains from CsCOL5–8 and AtCOL9–15 in Group III showed 74% and 60% identity, respectively. In particular, in the divergent B-box2 domain, only five out of 29 amino acids (17.2%) were fully conserved (Fig. 2D). The B-box1 domain showed 81% identity among CsCOL9 and AtCOL6/7/8/16 in Group II. Its consensus sequence was C-X2-C-X5-A-X-W-Y- C-X2-A-F-L-C-X2-C-D-X3-H-S-A-N- X2-A.

-X2-H, and 20 out of 38 (52.6%) amino acids were fully conserved (Fig. 2E). The CCT domain showed 76% identity among eight CsCOL and 17 AtCOL proteins, and 16 out of 42 (38%) amino acids were fully conserved (Fig. 2F). Thus, the most conserved domain was the B-box1 domain of CsCOL9 in Group II and the least conserved domain was the divergent B-box2 of CsCOL5-8 in Group III, relative to the AtCOLs domains.

### Subcellular localization of five CsCOL proteins

**Fig. 3:**
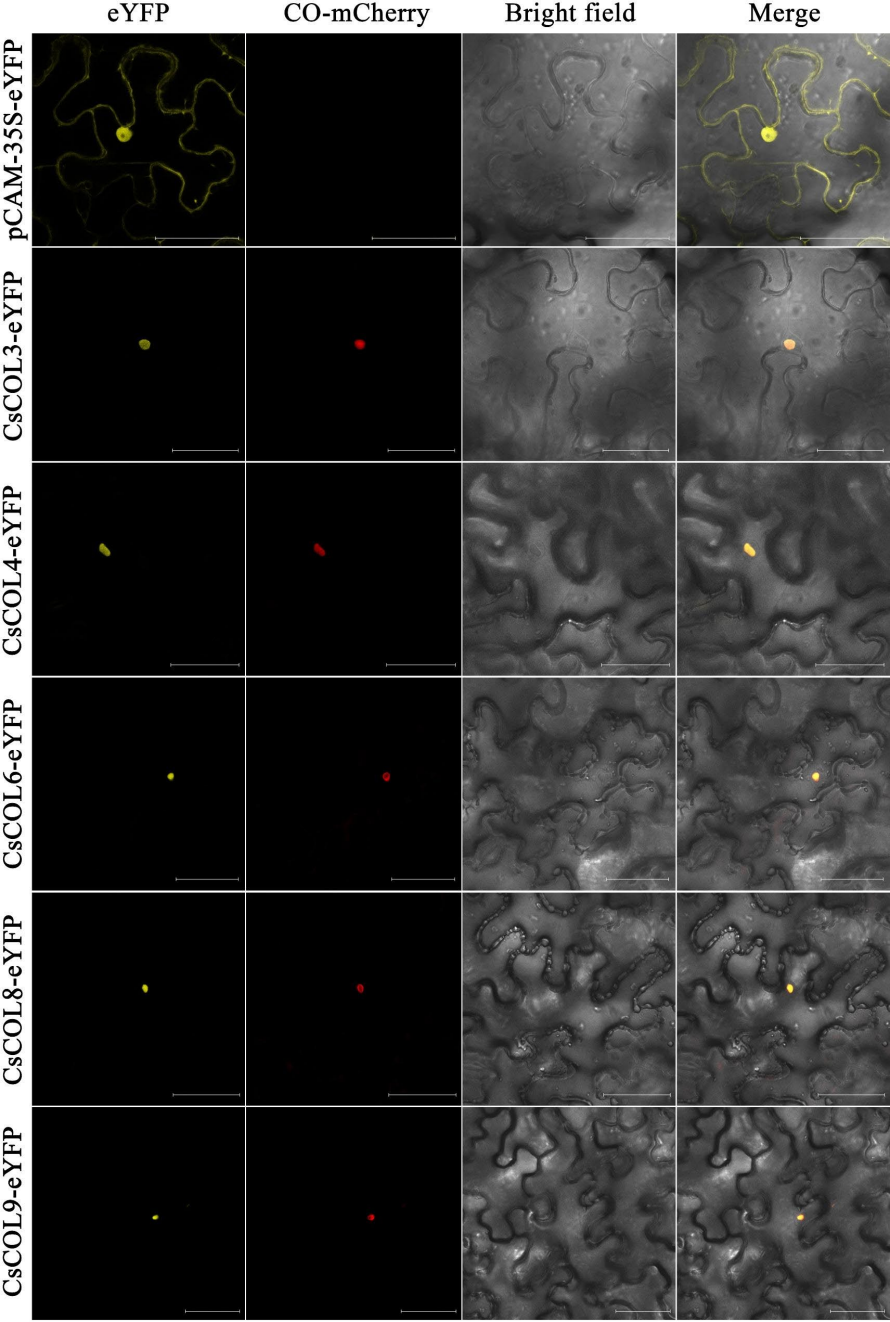
Subcellular localization of five CsCOL proteins (CsCOL3, CsCOL4, CsCOL6, CsCOL8, CsCOL9) in *Nicotiana tabacum* cells. The negative control is pCAM-35 S-YFP. The nuclear localization marker protein is AtCO-mCherry. Bars = 50 μm

To test whether CsCOL proteins are localized in the nucleus and whether the CCT domain lies near the C-terminus, five CsCOL proteins (CsCOL3/4/6/8/9) from three groups were selected to analyze their subcellular localization. For each, a translational fusion of yellow fluorescent protein (YFP) and CsCOL proteins (*35 S:YFP-CsCOL*) was constructed, and a nuclear localization marker (AtCO-mCherry) was used to identify the localization of CsCOL proteins using the transient expression system of *Nicotiana tabacum* epidermal cells. The five YFP-CsCOL fusion proteins were mainly detected in the nuclei (Fig. 3). The mCherry signal of the AtCO-mCherry nuclear localization marker overlapped with the signals of YFP-CsCOL3/4/6/8/9, indicating that CsCOL3/4/6/8/9 were clearly localized in the nucleus of *N. tabacum* epidermal cells, similar to AtCO from *A. thaliana* [6] and *PhalCOL* from *Phalaenopsis hybrida* [24]. Based on this finding, we conclude that CsCOL3/4/6/8/9 are nucleus-localized proteins.

### Expression patterns of five CsCOL genes in various organs and developmental stages

**Fig. 4:**
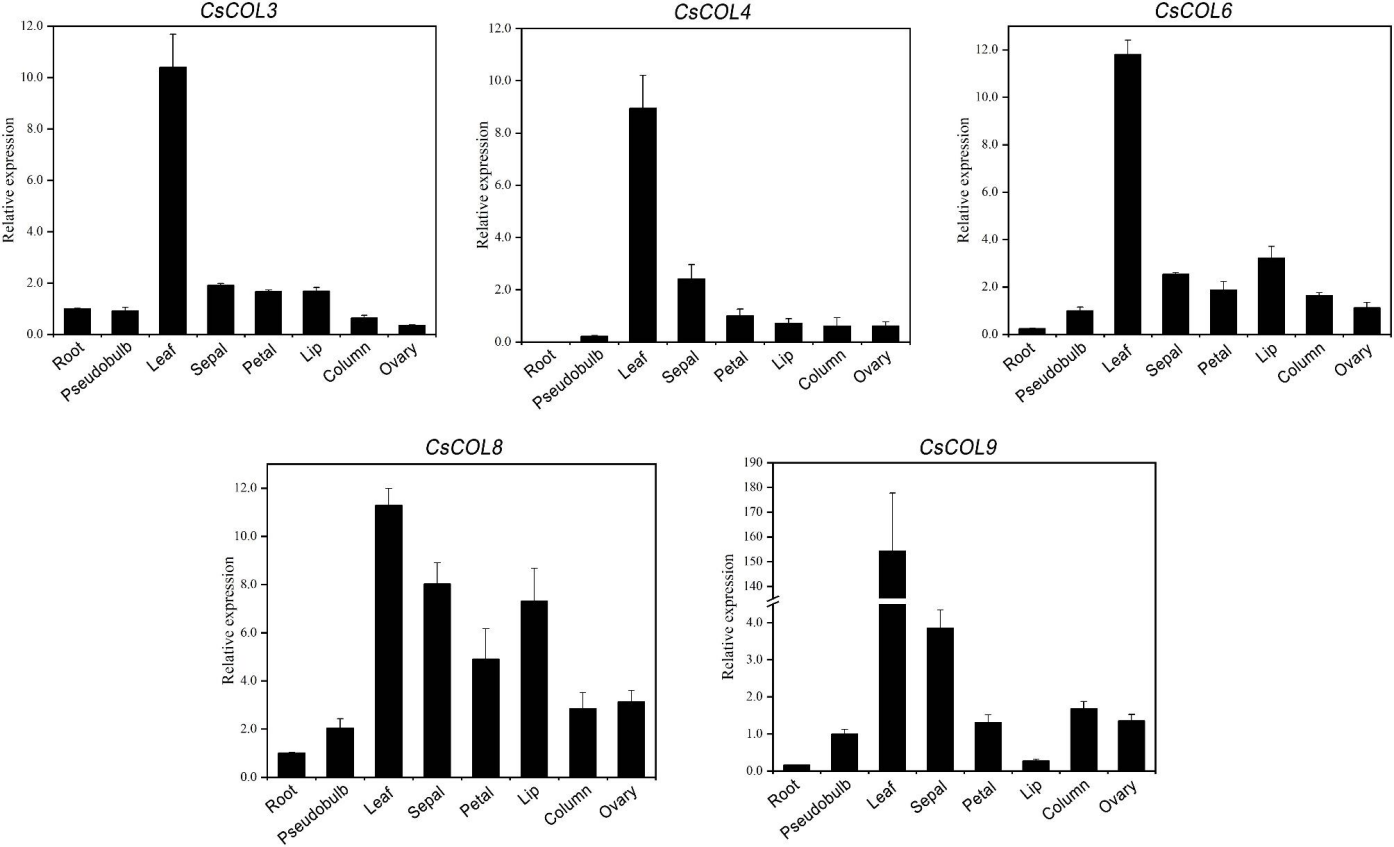
qRT-PCR-based analysis of the expression of five *CsCOL* genes in different organs of *C. sinense*. Data bars represent the mean ± SD of three biological replicates

Five *CsCOL* genes (*CsCOL3/4/6/8/9*) from three groups were selected to analyze their tissue-specific expression patterns by qRT-PCR. The five *CsCOL* genes were detected in almost all organs (roots, pseudobulbs, leaves, sepals, petals, lips, columns and ovaries) at the initial flowering stage. Five *CsCOL* genes were mainly expressed in the leaves, and the lowest expression was in roots (Fig. 4). Apart from their high expression levels in leaves, the expression of *CsCOL* genes was also high in floral organs, particularly in sepals or lips (Fig. 4).

To comprehensively compare the expression levels of the five *CsCOL* genes in various organs, a heat map was constructed based on qRT-PCR results. The result was shown in Figure S1. The expression of *CsCOL3* in roots was set to 1, and the relative expression of other genes was then adjusted. A visual gene expression profile was generated by TBtools software [25]. Based on a color code, *CsCOL3*/*4* in Group I were more highly expressed in all organs than other *CsCOL* genes. *CsCOL6*/*8* in Group III were mainly expressed in leaves, but their expression levels were lower than those of *CsCOL3/4* in Group I while *CsCOL9* in Group II was specifically and highly expressed in leaves, relative to other organs (Figure S1).

**Fig. 5:**
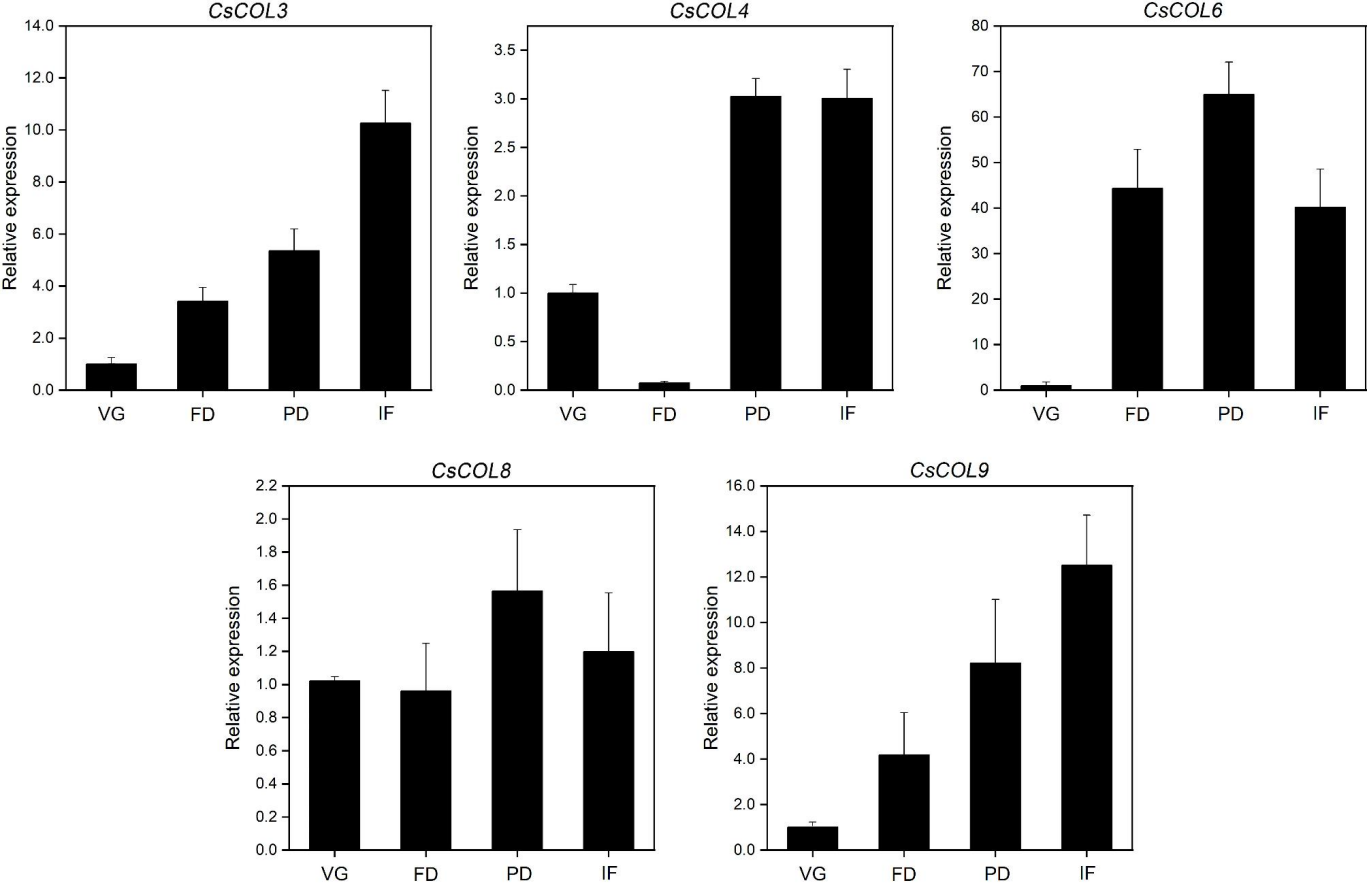
Relative expression of five *CsCOL* genes in the leaves at four developmental stages of *C. sinense*. VG, vegetative growth stage; FD, flower bud development stage; PD, pedicel development stage; IF, initial flowering stage. Data bars represent the mean ± SD of three biological replicates

To analyze the expression patterns of the five *CsCOL* genes in different developmental stages, their expression levels were detected in four representative developmental stages: vegetative growth (VG), flower bud differentiation (FD), pedicel development (PD), and initial flowering (IF) (Fig. 5). The expression levels of *CsCOL3/4/9* gradually increased from VG to IF, and peaked at the IF stage, except for the lowest expression levels of *CsCOL4* at the FD stage. Highest expression of *CsCOL6/8* was at the PD stage, but it began to decrease in IF. Moreover, *CsCOL6* was barely expressed during VG but was strongly expressed at other floral developmental stages. These results suggest that these five *CsCOL* genes might play different roles in the different floral developmental stages of *C. sinense*.

### Expression patterns of five ***CsCOL*** genes in different photoperiods

**Fig. 6:**
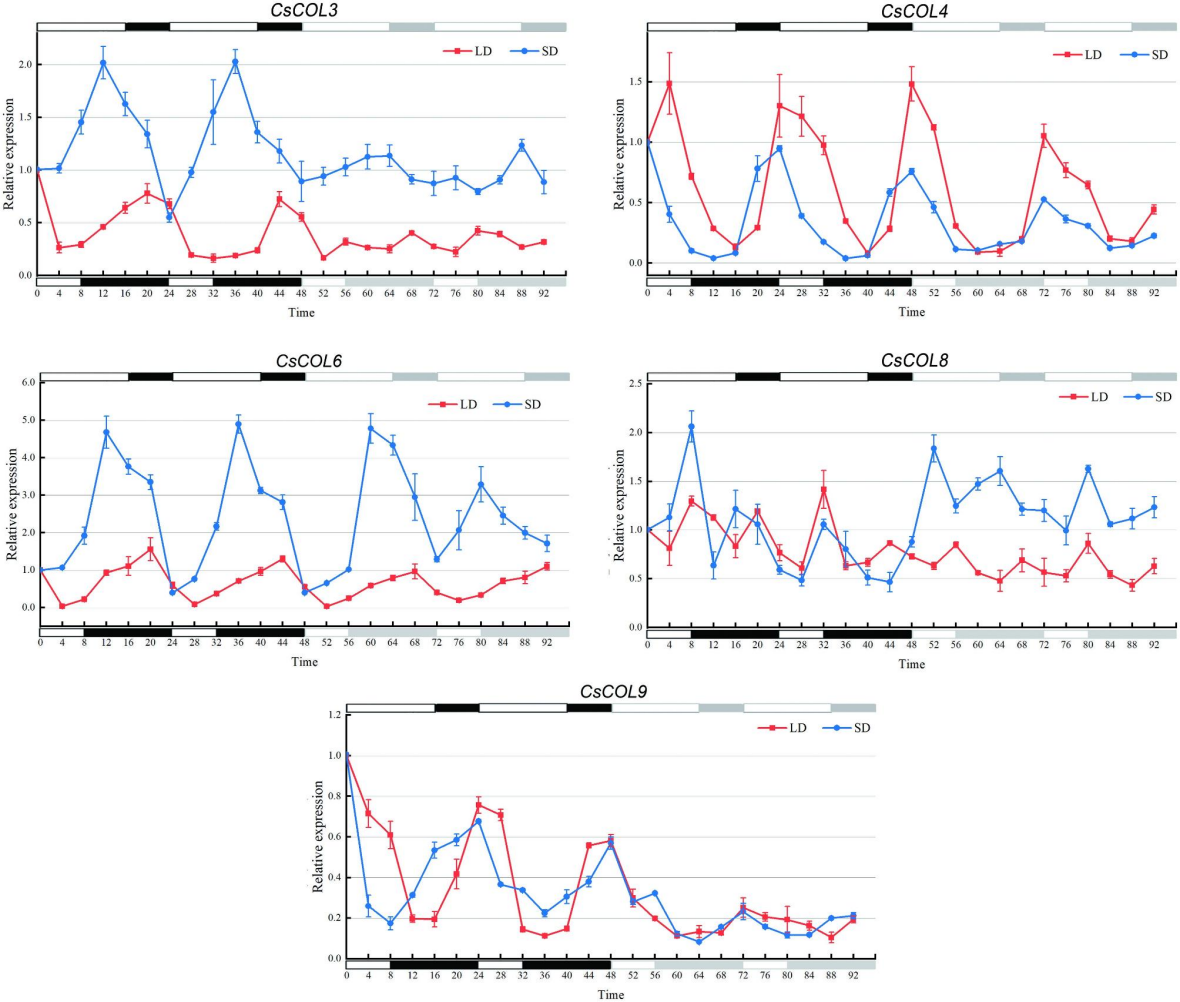
Circadian rhythm-induced expression patterns of five *CsCOL* genes in long days (LD) and short days (SD). White represents light and black represents darkness. After 48 h of treatment, plants were exposed to 48 h of continuous light (CL). Young leaves were sampled every 4 h over a 96-h period. Data bars represent the mean ± SD of three biological replicates

To further study the photoperiodic rhythm of the five *CsCOL* genes, their expression patterns in leaves in different photoperiods were analyzed by qRT-PCR. As shown in Fig. 6, the diurnal oscillation of the five *CsCOL* genes exhibited three patterns after LD or SD treatment. *CsCOL3/6* expression exhibited similar diurnal fluctuations and showed a single peak in the first 24 h in LD, and was lowest after 4 h of light, but peaked after 4 h of darkness, then gradually decreased in the first 24-h period. Circadian expression in the second 24-h period was similar to that in the first 24-h period. *CsCOL3*/*6* expression patterns during 48 h in constant light were similar to their response in LD. *CsCOL3*/*6* expression in SD also exhibited similar diurnal fluctuations and showed a single peak in the first 24 h. Their expression increased in light. The peak occurred after roughly 4 h of darkness then decreased until 24 h after dawn. In the subsequent 48 h of constant light, fluctuations in expression repeated the pattern in SD. *CsCOL3*/*6* expression was higher in SD than in LD, suggesting that *CsCOL3*/*6* was strongly induced in SD relative to LD. The circadian expression patterns of *CsCOL4/9* showed no significant differences between LD and SD. Their expression was repressed in light, lowest at dusk, and showed a dramatic increase in the dark, peaking at dawn. In continuous light, the rhythm of *CsCOL4/9* expression was repeated, similar to LD or SD. These results suggest that the diurnal expression rhythm of *CsCOL4/9* was not affected by the duration of light. The expression of *CsCOL8* in SD exhibited diurnal fluctuations and peaked twice in a 24-h period. The expression was gradually up-regulated and peaked initially at dust while the second peak occurred at 16 h in the dark in SD. The expression of *CsCOL8* in LD was more erratic, and its expression level was higher in SD than in LD in the subsequent 48 h of constant light. The results suggest that *CsCOL/3/4/6/8/9* had different response mechanisms and functions to photoperiodic regulation in *C. sinense.*

### Overexpression of ***CsCOL3/4/6/8*** in ***A. thaliana*** affect flowering and growth

The phylogenetic analysis shown that CsCOL3/4 were grouped with CsCOL1 in Group I (Fig. 2). *CsCOL1* promotes early flowering in *A. thaliana* (Zhang et al., 2020). CsCOL5/6/7/8 were grouped with AtCOL9 in Group III (Fig. 2). *AtCOL9* delays flowering in LD (Cheng et al., 2005). Moreover, the second B-box domain was different between CsCOL3 and CsCOL4, and between CsCOL6 and CsCOL8 (Fig. 1). Consequently, we first selected *CsCOL3/4/6/8* to analyze their biological functions. The *35 S::CsCOL* vectors were constructed and transformed into *A. thaliana*, and three independent transgenic T2 generation plants were randomly selected to examine their flowering time in LD and SD. We confirmed that the transgenic lines carrying an empty vector did not differ significantly from wild type (WT) plants. All transgenic lines showed high expression levels of *CsCOL*3/4/6/8 in *A. thaliana* (Figure S2).

**Fig. 7:**
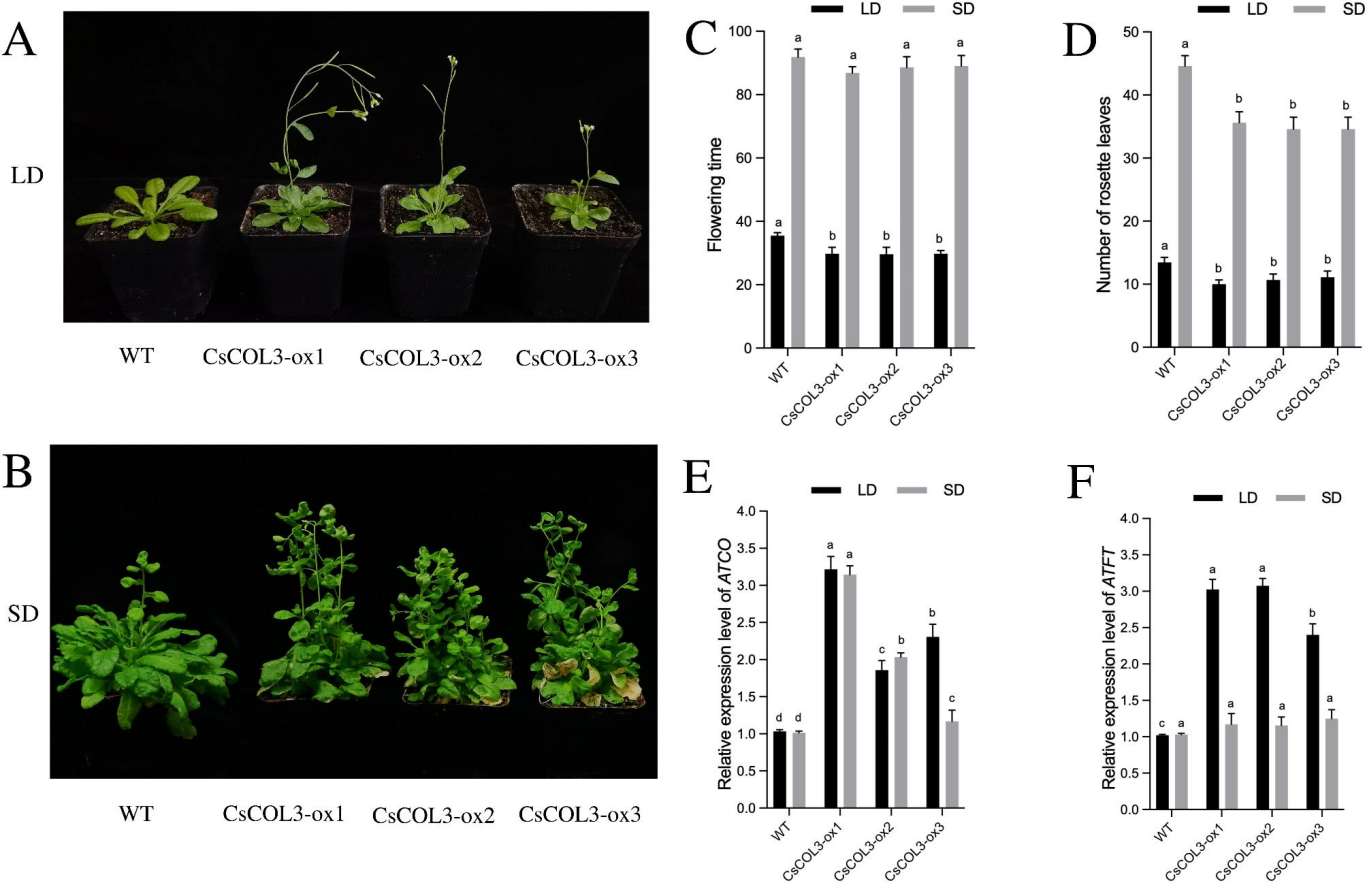
Functional analysis of *CsCOL3* in transgenic *Arabidopsis thaliana* plants in LD and SD. (**A**) Flowering phenotypes in LD, (**B**) Flowering phenotypes in SD, (**C**) flowering time, (**D**) number of rosette leaves, (**E**) *AtCO* expression levels of wild-type (WT) and three *35 S::CsCOL3* transgenic *A. thaliana* plants (*CsCOL3-ox1/2/3*). (**F**) *AtFT* expression levels of wild-type (WT) and three *35 S::CsCOL3* transgenic *A. thaliana* plants (*CsCOL3-ox1/2/3*). Data bars represent the mean ± SD of three biological replicates. Asterisks indicate significant differences (at *P* < 0.05; student’s *t*-test) relative to WT. Bar = 1 cm

Three *CsCOL3* transgenic lines (*CsCOL3-ox1/2/3*) showed an early flowering phenotype under LD, with shortened flowering time (Fig. 7A and C). However, overexpression *CsCOL3* in *A. thaliana* had no significant effect on flowering time in SD, but could induce more inflorescences (Fig. 7B and C). Compared with WT plants, the number of rosette leaves in transgenic lines decreased significantly under both LD and SD (Fig. 7D). The expression level of *ATCO* was significantly upregulated in *35 S::CsCOL3* transgenic plants under both LD and SD, relative to WT (Fig. 7E). The expression level of *ATFT* in *35 S::CsCOL3* transgenic plants was also significantly upregulated under LD, but not significantly higher in *35 S::CsCOL3* transgenic plants than in WT under SD (Fig. 7F).

**Fig. 8:**
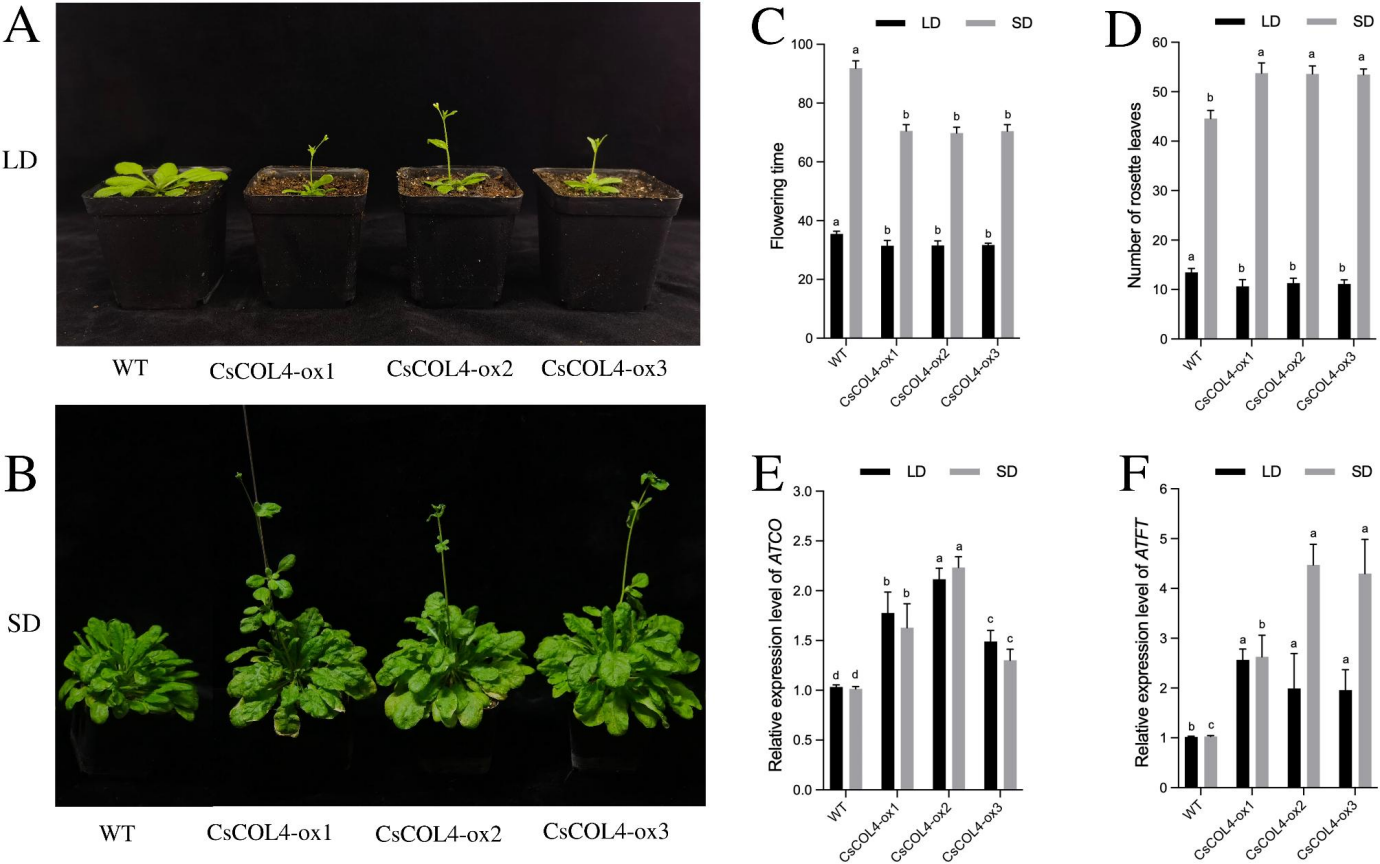
Functional analysis of *CsCOL4* in transgenic *Arabidopsis thaliana* plants in LD and SD. (**A**) Flowering phenotypes in LD, (**B**) Flowering phenotypes in SD, (**C**) flowering time, (**D**) number of rosette leaves, (**E**) *AtCO* expression levels of wild-type (WT) and three *35 S::CsCOL4* transgenic *A. thaliana* plants (*CsCOL4-ox1/2/3*). (**F**) *AtFT* expression levels of wild-type (WT) and three *35 S::CsCOL4* transgenic *A. thaliana* plants (*CsCOL4-ox1/2/3*). Data bars represent the mean ± SD of three biological replicates. Asterisks indicate significant differences (at *P* < 0.05; student’s *t*-test) relative to WT. Bar = 1 cm

The *35 S::CsCOL4* transgenic lines (*CsCOL4-ox1/2/3*) had an early flowering phenotype under both LD and SD (Fig. 8A and B). Compared to WT, the flowering time was reduced significantly in transgenic plants under LD and SD (Fig. 8C). The number of rosette leaves during bolting in transgenic plants was reduced significantly under LD, but increased under SD (Fig. 8D). The expression level of *AtCO* and *AtFT* increased significantly in *35 S::CsCOL4* transgenic *A. thaliana* under both LD and SD (Fig. 8E and F).

**Fig. 9:**
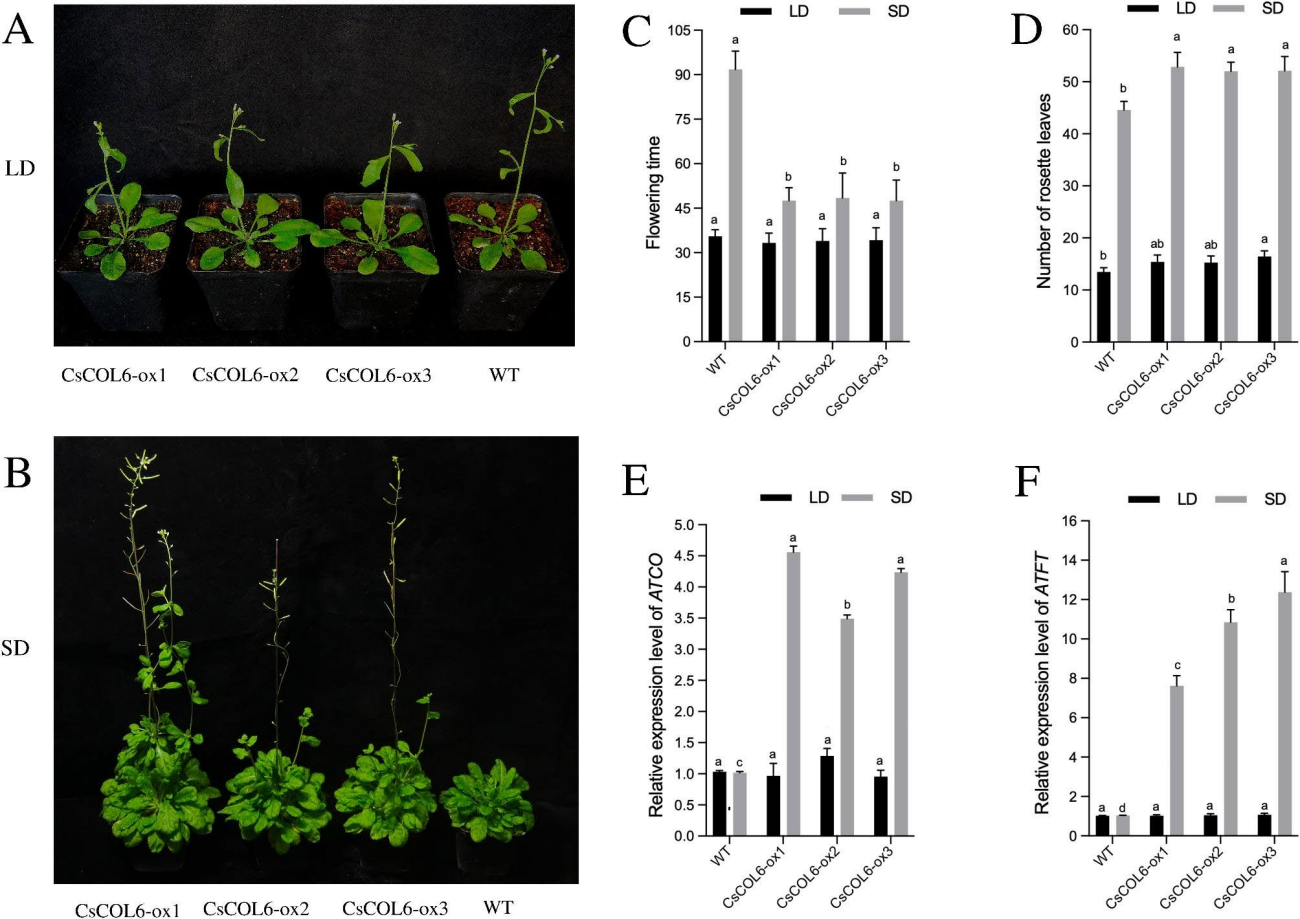
Functional analysis of *CsCOL6* in transgenic *Arabidopsis thaliana* plants in LD and SD. (**A**) Flowering phenotypes in LD, (**B**) Flowering phenotypes in SD, (**C**) flowering time, (**D**) number of rosette leaves, (**E**) *AtCO* expression levels of wild-type (WT) and three *35 S::CsCOL6* transgenic *A. thaliana* plants (*CsCOL6-ox1/2/3*). (**F**) *AtFT* expression levels of wild-type (WT) and three *35 S::CsCOL6* transgenic *A. thaliana* plants (*CsCOL6-ox1/2/3*). Data bars represent the mean ± SD of three biological replicates. Asterisks indicate significant differences (at *P* < 0.05; student’s *t*-test) relative to WT. Bar = 1 cm

Overexpression of *CsCOL6* in transgenic *A. thaliana* did not promote early flowering under LD (Fig. 9A), but displayed early flowering phenotype under SD (Fig. 9B). The flowering time and the number of rosette leaves in transgenic plants were also no significant difference between *35 S::CsCOL6* transgenic *A. thaliana* with WT plants under LD (Fig. 9C and D). While under SD, the flowering time reduced, and the number of rosette leaves during bolting increased significantly in transgenic plants (Fig. 9C and D). The expression level of *AtCO* and *AtFT* were significantly up-regulated in *35 S::CsCOL6* transgenic *A. thaliana* under SD (Fig. 9E and F), consistent with their phenotypic results.

**Fig. 10:**
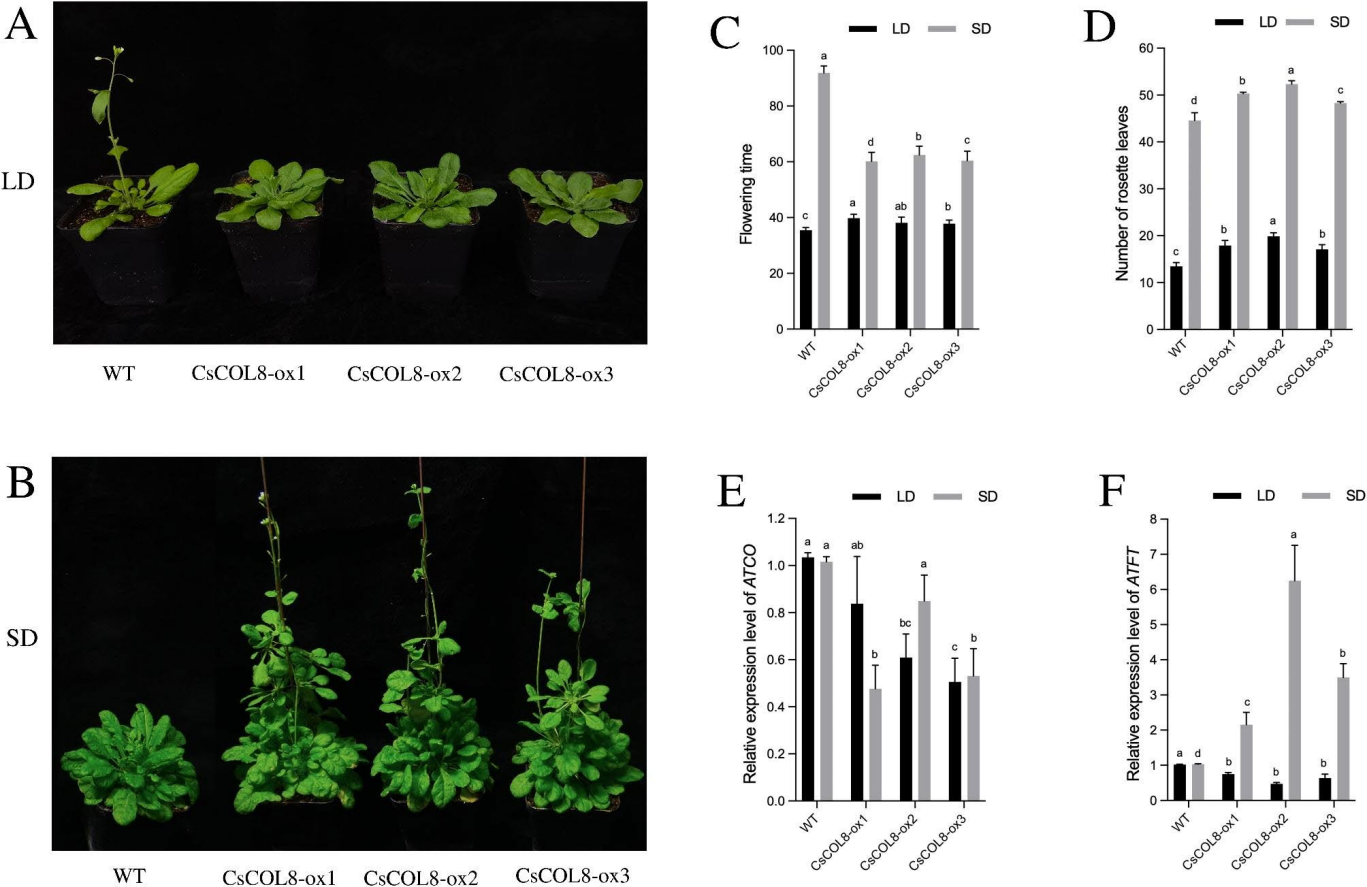
Functional analysis of *CsCOL8* in transgenic *Arabidopsis thaliana* plants in LD and SD. (**A**) Flowering phenotypes in LD, (**B**) Flowering phenotypes in SD, (**C**) flowering time, (**D**) number of rosette leaves, (**E**) *AtCO* expression levels of wild-type (WT) and three *35 S::CsCOL8* transgenic *A. thaliana* plants (*CsCOL8-ox1/2/3*). (**F**) *AtFT* expression levels of wild-type (WT) and three *35 S::CsCOL8* transgenic *A. thaliana* plants (*CsCOL8-ox1/2/3*). Data bars represent the mean ± SD of three biological replicates. Asterisks indicate significant differences (at *P* < 0.05; student’s *t*-test) relative to WT. Bar = 1 cm

Overexpression of *CsCOL8* in transgenic *A. thaliana* showed different flowering phenotype under LD and SD. Overexpression of *CsCOL8* showed a late flowering phenotype in LD (Fig. 10A), but promoted earlier flowering in SD (Fig. 10B). Compared with WT plants, the flowering time in transgenic lines increased under LD and reduced under SD (Fig. 10C). However, the number of rosette leaves in transgenic lines increased under both LD and SD (Fig. 10D). The expression levels of *AtCO* was decreased in *35 S::CsCOL8* transgenic plants both under LD and SD, relative to WT (Fig. 10E). But the expression levels of *AtFT* only increased under SD (Fig. 10F).

## Discussion

*C. sinense* is a high-grade traditional potted flowering plant in China. The floral development process lasts about half a year and is regulated by multiple factors, including temperature, light and hormones. To dissect the molecular regulation of the photoperiodic flowering pathway, we identified and characteried *CO*/*COL* family genes in *C. sinenese*.

### Diverse characterization of ***CsCOL*** family members based on phylogeny and function

It has already been reported that *CO*/*COL* family genes play an important role in the photoperiod pathway. This family includes a wide diversity of members in different plant species, such as 17 members in *A. thaliana* [7], 16 in *O. sativa* [26], 9 in *H. vulgare*, 26 in soybean [27], 11 in *C. lavandulifolium* [28] and 8 in Asiatic hybrid lily [29]. In this study, eight *CsCOL* genes were identified based on an unpublished *C. sinense* transcriptomic database. A phylogenetic tree of *COL* homologs among *C. sinense*, *A. thaliana*, *O. sativa*, *Z. mays*, and *H. vulgare* was constructed. They were clustered into three groups based on the number and structure of their B-box domains, similar to the *CO* family in *A. thaliana* [7]. *CsCOL1/3/4* clustered in Group I. Some studies have shown that the *COL* homologs in Group I might have an inducing effect on flowering [24, 30, 31]. *CsCOL1* has already been reported as a floral inducer [32]. *CsCOL3/4* promoted early flowering in transgenic *A. thaliana*, and even though CsCOL3/4 were clustered in Group I in the phylogenetic tree, their B-box domains are different (Fig. 1). The expression pattern at the four developmental stages and photoperiodic rhythm of *CsCOL3* was also different from that of *CsCOL4* (Fig. 5). These results suggest that *CsCOL3/4* might act as flowering activators and play important roles at the different floral developmental stages. CsCOL5/6/7/8 clustered together in Group III, but the structure of B-box2 of CsCOL8 was different from that of CsCOL6. Perhaps their structural differences caused a functional diversity during flowering.

### CsCOL family members may possess various functions during floral development

*COL* homologs are broadly expressed throughout the life cycle of plants from the vegetative phase to the reproductive phase, such as *VvCO* and *VvCOL1* in latent buds of *Vitis vinifera* [33], *AtCOL8* in seeds, leaves, flowers, and siliques [13], as well as *PhalCOL* in inflorescences of *Phalaenopsis hybrida* [24]. In this study, five *CsCOL* genes showed differential expression patterns in all organs and at different developmental stages. The highest expression levels of five *CsCOL* genes was in leaves and the lowest in roots, similar to *ClCOL10* from *C. lavandulifolium* [28] and *PhalCOL* from *Phalaenopsis* [24]. Given that leaves are the most important photosynthetic organs of plants since they receive light signals [34], COL in leaves may sense daylength and perceive photoperiod. Apart from their high expression levels in leaves, the expression of five *CsCOL* genes was also high in floral organs, particularly in sepals and lips (Fig. 4), similar to the expression of *DcCOL* in *Dendrobium crumenatum* Swartz [35] and *BtCO* in bamboo [36], but unlike *PhalCOL* expression in *Phalaenopsis*, in which expression was particularly intense in pollinaria [24]. Sepals, which are the largest floral organ in the outermost layer of *C. sinense*, receive light for a longer period and over a larger area than other floral tissues. These results suggest that these five Cs*COL* genes might be involved in the morphogenesis of floral organs.

The entire floral developmental process of *C. sinense* lasts about half a year, mainly contains the flower bud differentiation, flower bud dormancy, pedicel development and flowering stages. Photoperiod had different effects on floral development at different stages. The expression levels of *CsCOL3*/*4*/*9* gradually increased from the vegetative growth stage to the initial flowering (IF) stage and showed highest expression in the IF stage, suggesting that *CsCOL3/4/9* might play an important role in floral development and promote flowering. However, the expression of *CsCOL4* was weak at the FD stage, but stronger at PD and IF stages, so it is likely involved in flowering regulation mainly in the late stage of inflorescence development. *CsCOL6/8* expression peaked in PD and decreased in IF, suggesting that they might play important roles only in pedicel inflorescence development, with weak functions during flowering. Flower buds differentiate from pseudobulbs. The levels of expression of five *CsCOL* genes (*CsCOL3*/*4*/*6/8/9*) in pseudobulbs and in FD were not high, suggesting their involvement in regulating flowering, primarily in the inflorescence development stage and not in the flower bud differentiation stage.

### CsCOL family members showed functional diversity in the photoperiodic flowering pathway

*CO* is a key gene in the photoperiodic pathway, but not all *CO/COL* family genes are regulated by photoperiod. The expression of *CsCOL3/4/6/8/9* showed different photoperiodic rhythms. *CsCOL3/6*, although belonging to Group I and II, respectively, showed similar diurnal rhythms of expression in LD and SD. Their expression levels in SD were higher than in LD, suggesting that *CsCOL3/6* might be strongly induced in SD relative to LD. The expression of *CsCOL4/9* was repressed in light, induced in the dark, and peaked at the end of the dark period, indicating that they may be involved in measuring the length of the dark period. The diurnal rhythms of *CsCOL4/9* were similar to those of *ClCOL1/2* in *C. lavandulifolium* [28], *AtCOL1/2* in *A. thaliana* [11], and *VvCO* and *VvCOL1* in *V. vinifera* [33]. However, the functions of these COL homologs are more diversified. *CsCOL8* exhibited diurnal fluctuations in SD, but showed no rhythmicity in LD. Moreover, *CsCOL8* expression was higher in SD than in LD in the subsequent 48 h of constant light. *CsCOL8* might thus be induced by SD. Taken together, SD could induce the transcript levels of *CsCOL3/4/6/8/9*, relative to LD. Consequently, additional research is needed to elucidate their functions in the molecular mechanism of SD-inductive flowering in *C. sinenese.*

The functions of most *CO/COL* family members in *A. thaliana* have been well characterized. Most of them are involved in flowering regulation through the photoperiodic pathway. For example, *AtCO* is induced by photoperiod and promotes flowering in LD [6], and *AtCOL5* induced flowering in SD [37]. In our study, the overexpression of *CsCOL3/4* promoted early flowering in *A. thaliana* under LD (Figs. 7A and 8A). Under SD condition, the overexpression of *CsCOL3/4* also played a positive role in flower development. Compared with WT, overexpression of *CsCOL3* does not promote early flowering in *Arabidopsis* under SD, but can induce more inflorescences (Fig. 7B). While *CsCOL4* not only induced early flowering, but also the growth of rosette leaves (Fig. 8B). The expression level of *AtCO* and *AtFT* were significantly upregulated in *35 S::CsCOL4* transgenic *A. thaliana* under LD and SD (Fig. 8E and F). The expression pattern of *AtCO* and *AtFT* were consistent. Therefore, *CsCOL4* might function as floral inducers in *C. sinense*, and could be actively involved in the *CO-FT* pathway, similar to *CsCOL1* [32]. The expression pattern of *AtCO* and *AtFT* in *35 S::CsCOL3* transgenic *A. thaliana* in LD was consistent, but not consistent under SD. The expression level of *AtCO* was significantly increased, but it did stimulate the expression of FT in *35 S::CsCOL3* transgenic plants under SD (Fig. 7E and F). *CO* acts upstream of *FT*, but *FT* is not the only target gene for *CO*. *CO* could also control flowering through other flowering pathways. *CsCOL3* It may be involved in regulating other downstream flowering genes and promoting flower bud formation under SD.

*CsCOL6* and *CsCOL8* were clustered together in Group III. Previous studies indicated that the *COL* homologs in Group III did not seem to promote photoperiod-mediated flowering. For example, *AtCOL9* delayed *A. thaliana* flowering in LD by inhibiting the expression of *AtCO* and *FT* [12]. *AtCOL12* repressed flowering by inhibiting CO function and FT transcriptional activation [38]. In this study, *CsCOL6* had little effect on flowering time, while *CsCOL8* delayed flowering in *A. thaliana* under LD (Figs. 9 and 10). The expression of *CsCOL8* in LD was more erratic, thus *CsCOL8* might function as a flowering repressor in a photoperiod-independent flowering pathway under LD. Another possibility is that *CsCOL8* may perceive light signals, then promote photosynthesis, leading to an increase in vegetative growth and suppression of reproductive growth, thereby delaying flowering. Unexpectedly, *CsCOL6* and *CsCOL8* were strongly induced under SD. The overexpression of *CsCOL6* and *CsCOL8* promoted earlier flowering and the growth of rosette leaves under SD. These results suggested that SD might be induce *CsCOL6* and *CsCOL8* positively regulating the floral development process of *C.sinense.* The phenotype of early flowering was accompanied with corresponding changes in *ATFT* expression. However, the expression level of *ATCO* was inhibited in the *35 S::CsCOL8* transgenic plants under SD. The expression pattern of *ATCO* does not match the phenotype. So, *CsCOL8* might function on regulating flowering, independently of *CO* under SD.

## Conclusion

Eight *CsCOL* genes in *C. sinense* have been identified and cloned for the first time. They could be divided into three groups based on a phylogenetic analysis, showed considerable variation in developmental stage specific expression and photoperiodic rhythms. *CsCOL3/4* were considered as important floral inducers in the photoperiodic flowering pathway. SD induced the expression of *CsCOL6/8*. *CsCOL6* and *CsCOL8* could induce early flowering and rosette leaves growth in transgenic *A. thaliana* under SD. But under LD condition, CsCO*L8* delayed flowering and *CsCOL6* did not affect flowering time in transgenic *A. thaliana*. These results indicated that *CsCOL3/4/6/8* genes in *C. sinense* were involved in growth, development and flowering regulation through different photoperiodic pathway. This study will also be of benefit for regulating flowering in this orchid.

## Methods

### Plant material growth conditions and sampling

*C. sinense* “Qi Jian Bai Mo” is a famous traditional variety in China. Three-years-old *C. sinense* ‘Qi Jian Bai Mo’ potted plants were provided and cultivated by Prof. Duan Jun from South China Botanical Garden. They were maintained in a greenhouse at the South China Botanical Garden of the Chinese Academy of Sciences, in Guangzhou, China. *A. thaliana* (ecovar Columbia) plants were grown in the following conditions: LD, 23 °C, 60% relative humidity, and 150 μmol m^− 2^ s^− 1^.

To analyze the tissue-specific expression patterns of *CsCOL* genes in *C. sinense*, roots, pseudobulbs, leaves, sepals, petals, lips, columns and ovaries were sampled at the initial flowering (IF) stage. To analyze expression of *CsCOL* genes during floral development, leaves were sampled at four development stages: vegetative growth (VG), flower bud differentiation (FD), pedicel development (PD), and initial flowering (IF) stage. To study whether photoperiod regulates *CsCOL* genes, plants were placed in a light incubator (constant 27 °C) under two photoperiodic conditions: LD, consisting of 16 h light and 8 h darkness, and SD, consisting of 8 h light and 16 h darkness. Light was provided by 30 W fluorescent bulbs (Philips, Shanghai, China). As in a previous study [32], plants were kept in LD and SD for 4 weeks, in LD or SD for 2 d, and finally in continuous light for 2 d. During the last 96 h, young leaves were sampled from the third node (counting from the apex), as three independent biological replicates (i.e., independent plants) every 4 h. Total RNA was isolated for qRT-PCR expression analysis.

### Screening and cloning of CsCOL genes from ***C. sinense***

*CONSTANS-like* genes were screened from *C. sinense* transcriptomic database (Accession SRA058042) based on functional annotation of transcripts and analysis of sequence similarity. The eight primer pairs (Table S1.1) were designed from the 5’ and 3’ ends and used to clone the ORFs of *CsCOL* genes by semi-quantitative RT-PCR as in Zhang et al [32]. Eight *CONSTANS-like* genes were identified.

### Protein conservative domain analysis and phylogenetic analysis of the COL family

The identified eight *COL* genes (*CsCOLs*) were submitted to the ExPASY website (http://web.expasy.org/protparam/) for predictive analysis of protein MW, pI, grand average of hydropathicity (GRAVY) and aliphatic index. The CsCOL protein sequences were submitted to the NCBI Conserved Domain Database (https://www.ncbi.nlm.nih.gov/cdd/) for CCT and B-box domains analysis with an e-value threshold of 0.01. These domains were compared with reported *COL* family sequences from *A. thaliana*, *O. sativa*, and *H. vulgare*. A phylogenetic tree was constructed by the neighbor-joining method [39] using MEGA 11 software. The conserved B-box and CCT domains from *C. sinense* and *A. thaliana* were aligned using the DNAMAN program.

### RNA extraction and qRT-PCR

Total RNA was extracted from each tissue sample using the MAGE RNA Extraction Kit, including the removal of redundant polysaccharides, following the manufacturer’s protocol (Maygene Bio Inc., Guangzhou, China). RNA samples were treated with RNase-free DNase I (Takara Bio Inc., Kyoto, Japan) to remove residual genomic DNA (gDNA). The first cDNA strand was synthesized based on purified RNA using the HiScript III RT SuperMix system (Vazyme Bio Inc., Nanjing, China). For qRT-PCR, this cDNA was used as a template after 10-fold dilution with ddH_2_O. *CsCOL* gene-specific primers (Table S1.2), based on their coding sequences, were designed using the online website primer3 plus (http://www.primer3plus.com/). qRT-PCR reactions were performed as three independent biological replicates for each sample using the ChamQ™ SYBR® qPCR Master Mix (Vazyme Bio Inc.). *C. sinense ACTIN* (NCBI accession number: GU181353) was employed as the internal reference gene [32] to standardize cDNA concentration. Relative gene expression was calculated using the 2^−∆∆CT^ method [40].

### Subcellular localization analysis

The full-length cDNAs of *CsCOL3/4/6/8/9* were amplified and inserted into the pCambia1301 vector to generate *35 S::CsCOL-YFP* fusion constructs. The nuclear localization of *A. thaliana AtCO* fused with mCherry was used as a positive nucleus marker to generate the *35 S::AtCO-mCherry* construct [41]. Tobacco (*N. tabacum*) leaves were used for transient expression assays as described elsewhere [42]. A Zeiss LSM 510 Meta confocal microscope (Wetzlar, Hesse, Germany) was used to detect YFP fluorescence signal of *N. tabacum* leaf epidermal cells at 514 nm. Table S1.3 lists the primers used to generate the five CsCOL-YFP fusion constructs.

### Overexpression vector construction, and ***A. thaliana*** transformation and phenotypic analysis

The *CsCOL3/4/6/8* full-length cDNAs were amplified with specific primers attached to *Kpn*I and *Sal*I digestion sites. The resulting PCR fragments were digested by two restriction enzymes (*Kpn*I and *Sal*I, Takara, Qingdao, China) to generate sticky ends. The pCAMBIA1301 vector with a 35 S promoter was digested with *Kpn*I and *Sal*I to linearize the plasmid. *CsCOLs* and pCAMBIA1301 fragments were then ligated using homologous recombination using the ClonExpress One Step Cloning Kit (Vazyme Bio Inc.). Integration of the full-length cDNAs into the constructed recombinant plasmid was confirmed by digesting them with restriction enzymes then sequencing them (Tsingke, Beijing, China). The recombinant plasmid was transformed into *Agrobacterium tumefaciens* strain GV3101 and genetically transformed into *A. thaliana* (ecovar. Columbia) plants by a floral dip method [43]. The wild *A. thaliana* was provided by Prof Liu L. from South China Agricultural University [44]. All primers are listed in Table S1.4.

In vitro selection of transgenic *A. thaliana* plants was on agar-based Murashige and Skoog (MS) medium [45] containing 30 mg/L hygromycin (Roche, Basel, Switzerland). Hygromycin-resistant plants were transferred to soil and grown as in Zhang et al. [32].

As in Zhang et al. [32], “flowering phenotypes were assessed in homozygous T_2_ generation plants based on the number of rosette leaves at bolting time when the inflorescence was 1 cm long [46]. Using data from 20 individual plants, values are represented as the means ± standard deviation (SD). Significant differences between means in pairwise comparisons were analyzed by SPSS version 20 software with the student’s *t*-test (*P* < 0.05).

*CsCOL3/4/6/8* insertion and expression in the *A. thaliana* genome was confirmed by semi-quantitative RT-PCR analysis using the *CsCOL3/4/6/8* primers. The RT-PCR specific primers of *CsCOL3/4/6/8* are listed in Table S1.1. The expression levels of and *AtCO* and *AtFT* in WT and transgenic *A. thaliana* were evaluated by qRT-PCR, and *A. thaliana TUB2* served as the internal control. The gene-specific primers of *AtCO, AtFT* as well as *TUB2* were the same as in Zhang et al. [32].

### Electronic supplementary material

Below is the link to the electronic supplementary material.

**Supplementary Material 1: Table S1**. 1. Primers used for cloning the ORF of *CONSTANS-like* genes in *Cymbidium sinense*. 2. Primers used for gene expression analysis by qPCR. 3. Primers used to construct vectors for subcellular localization. 4. Primers used to construct the vectors for Overexpression Vector Construction

**Supplementary Material 2: Table S2**. The Sequences of 8 *CsCOL* genes were cloned in this study

**Supplementary Material 3: Table S3** Molecular information and subcellular localization prediction of *COL* gene family in *Cymbidium sinense*

**Supplementary Material 4: Figure S1**. Expression of *CsCOLs* genes in different organs of root, pseudobulb, leaf, sepal, petal, lip, column and ovary at the initial flowering stage. The heatmap was created by Tbtools based on the transformed data of log2 (FPKM+1) values and the cluster analysis was performed on gene expression level by row. Expression differences are shown in different colors. Red means high expression and blue means low expression

**Supplementary Material 5: Figure S2**. Expression levels of *CsCOL3/4/6/8* in three *35S::CsCOL* transgenic *Arabidopsis* (L1/L2/L3) and WT were determined using semi-quantitative RT-PCR analyses and gel electrophoresis in 20-day-old WT and transgenic seedlings. The expression results were normalized against *A. thaliana TUB2* expression. (A) the expression levels of *CsCOL3*, (B) the expression levels of *CsCOL4*, (C) the expression levels of *CsCOL6*, (D) the expression levels of *CsCOL8*

**Supplementary Material 6: Figure S3**. The original gel electrophoresis image for Semi-quantitative PCR (RT-PCR) expression analyses of *CsCOL3/4/6/8* in three *35S::CsCOL* transgenic *Arabidopsis* (Ll/L2/L3) and WT. *A. thaliana TUB2* as the internal reference gene.

## Data Availability

All data analysed during this study are included in the supplementary information files. These genes have been deposited to NCBI, and their GenBank accession numbers are GU168786, OR526963, OR526964, OR526965, OR526966, OR526967, OR526968, OR526969.
