# Supplementary figures and images for: Identification of the CONSTANS-like family in Cymbidium sinense, and their functional characterization
Source: BMC Genomics. 2023 Dec 18;24:786. doi: 10.1186/s12864-023-09884-3 (PMC10729429; doi:10.1186/s12864-023-09884-3)

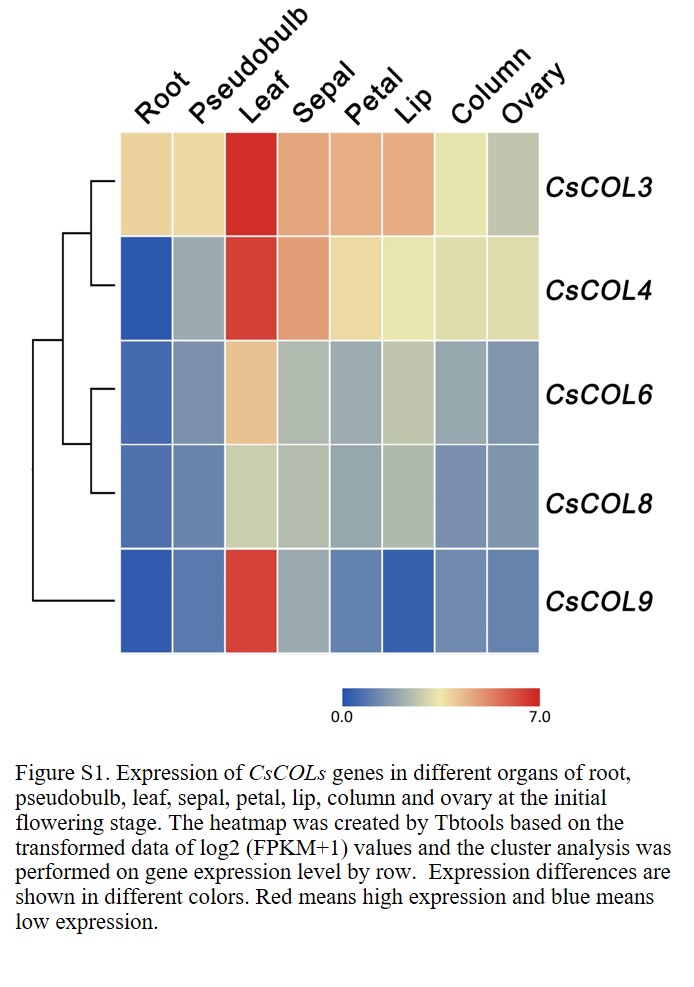

Supplement: Supplementary file 4 — Supplementary Material 4: Figure S1. Expression of CsCOLs genes in different organs of root, pseudobulb, leaf, sepal, petal, lip, column and ovary at the initial flowering stage. The heatmap was created by Tbtools based on the transformed data of log2 (FPKM+1) values and the cluster analysis was performed on gene expression level by row. Expression differences are shown in different colors. Red means high expression and blue means low expression [file 12864_2023_9884_MOESM4_ESM.jpg]

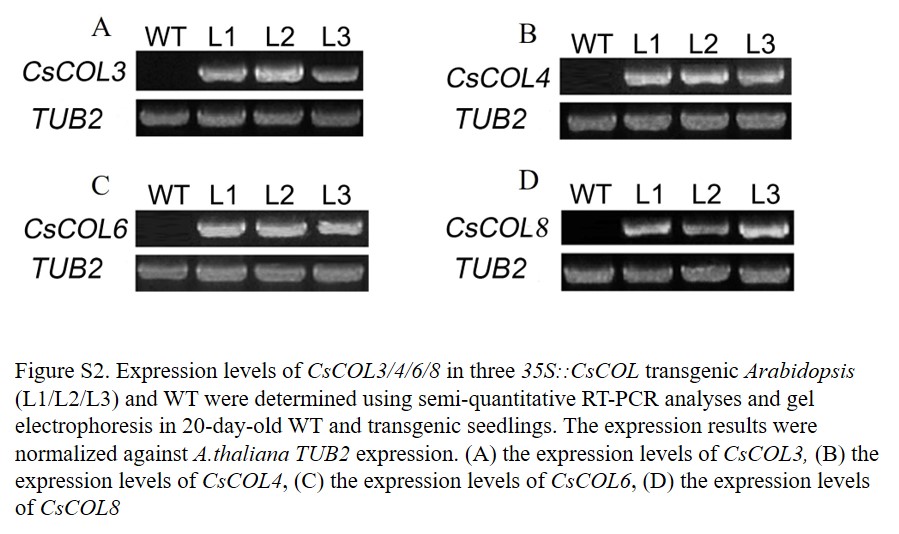

Supplement: Supplementary file 5 — Supplementary Material 5: Figure S2. Expression levels of CsCOL3/4/6/8 in three 35S::CsCOL transgenic Arabidopsis (L1/L2/L3) and WT were determined using semi-quantitative RT-PCR analyses and gel electrophoresis in 20-day-old WT and transgenic seedlings. The expression results were normalized against A. thaliana TUB2 expression. (A) the expression levels of CsCOL3, (B) the expression levels of CsCOL4, (C) the expression levels of CsCOL6, (D) the expression levels of CsCOL8 [file 12864_2023_9884_MOESM5_ESM.jpg]

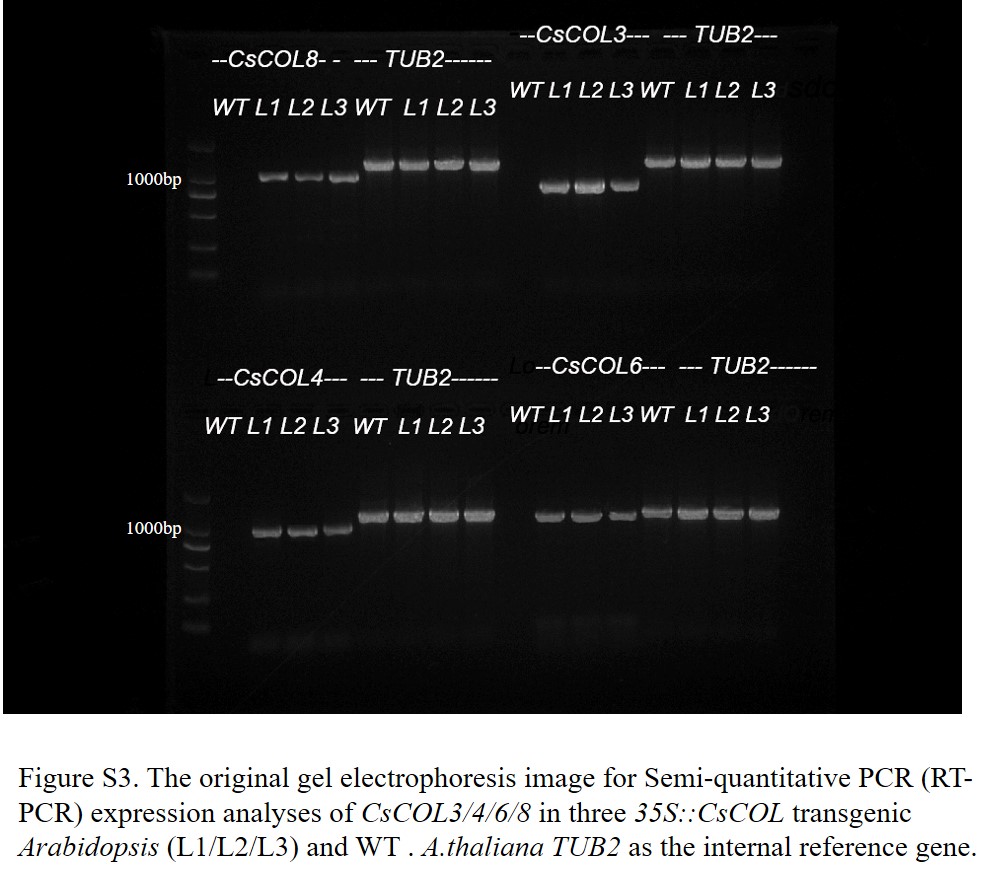

Supplement: Supplementary file 6 — Supplementary Material 6: Figure S3. The original gel electrophoresis image for Semi-quantitative PCR (RT-PCR) expression analyses of CsCOL3/4/6/8 in three 35S::CsCOL transgenic Arabidopsis (Ll/L2/L3) and WT. A. thaliana TUB2 as the internal reference gene. [file 12864_2023_9884_MOESM6_ESM.jpg]
